# Case report - Bouveret’s syndrome with pancreatitis: A rare combination

**DOI:** 10.1016/j.ijscr.2021.105713

**Published:** 2021-02-27

**Authors:** Wei Shearn Poh, Ruwan Wijesuriya

**Affiliations:** General Surgery, St John of God Midland Hospital, 1 Clayton Street, Midland, 6056 Western Australia, Australia

**Keywords:** Bouveret’s syndrome, Gastric outlet obstruction, Gallstone ileus, Cholecystoduodenal fistula, Pancreatitis, Case report

## Abstract

**Introduction and importance:**

Bouveret’s syndrome is characterised by gastric outlet obstruction due to impaction of gallstone in the duodenum through a cholecystoduodenal fistula, having concurrent pancreatitis makes it an even rarer presentation.

**Case presentation:**

We present an 82-year-old woman who presented with acute pancreatitis which she described 2 days of right upper quadrant pain with nausea and vomiting. Biliary obstruction signs were not present. She was known to have cholelithiasis but was for non-operative management due to significant cardiac history and multiple comorbilities.

**Clinical findings and investigations:**

On initial examination, abdomen was soft with mild right upper quadrant tenderness. Murphy sign was negative. Lipase level was raised at 64,261U/L with cholestasis appearance on liver function test. Bouveret’s syndrome was later diagnosed on CT after symptoms of gastric outlet obstruction surfaced during her admission.

**Interventions and outcome:**

An on-table endoscopic stone retrieval was done as first-line treatment. After multiple attempts of stone retrieval via endoscopy, surgical extraction of the impacted gallstone was required. Patient’s post-operative care was further challenged by effects from pancreatitis. Patient eventually recovered well and was discharged from hospital.

**Relevance and impact:**

When managing patients with gallstone pancreatitis, presence of persistent vomiting should raise suspicion of Bouveret’s syndrome. Bouveret’s syndrome can be diagnosed in these patients with aid of CT imaging or endoscopy. Stone extraction is required to treat Bouveret’s syndrome, endoscopic retrieval is first-line therapy as surgery has mortality risk of 12–30%. Prolonged recovery period can be expected due to concurrent inflammatory effects from acute pancreatitis.

## Highlights

• High suspicion of Bouveret’s syndrome required in pancreatitis with persistent vomit.
• CT imaging or endoscopy are suitable tools to diagnose Bouveret’s syndrome.
• Endoscopy retrieval is first-line therapy as surgical mortality risk is 12–30%.
• 90% of cases require surgical stone retrieval if cannot be removed endoscopically.
• Prolonged recovery can be expected as pancreatitis complicates patient’s management.

## 1 Introduction

Bouveret’s syndrome is a rare form of gastric outlet obstruction caused by impaction of a gallstone within the duodenum after migrating via a cholecystoduodenal fistula [1]. It was first described by Leon Bouveret in 1896 [2]. It accounts for 1–3% of gallstone ileus cases [3]. Endoscopy can be a useful diagnostic and therapeutic tool for identifying and extracting the stone, however majority of cases require surgical treatment [[3], [4], [5], [6], [7], [8]]. Presence of concurrent acute pancreatitis is an even rare presentation which adds further complexity as a result of systemic inflammatory response and technical difficulty in duodenal surgery from pancreatic inflammation [[8], [9], [10], [11]]. The following case report is presented in line with the SCARE criteria [12].

## 2 Case presentation

An 82-year-old woman presented to the emergency department with 2-day history of right upper quadrant pain with associated nausea and vomiting. She denies signs of biliary obstruction and fever. She was known to have cholelithiasis on previous ultrasound and was for non-operative management due to significant comorbidities including ischaemic heart disease/myocardial infarction, congestive heart failure, cardiac defibrillator in situ, type 2 diabetes on insulin, hypertension, and obesity with BMI 35. On examination, the abdomen was soft with mild tenderness in the right upper quadrant with negative Murphy’s sign. Blood investigations found that she had a significantly raised lipase of 64,261U/L with liver function test showing cholestasis. An ultrasound of the abdomen was performed as an initial assessment, as MRCP and CT cholangiogram were contraindicated due to presence of cardiac defibrillator and bilirubin at 58umol/L respectively, which showed large calculi up to 33 mm within the gallbladder. The biliary tree was not dilated.

**Fig. 1:**
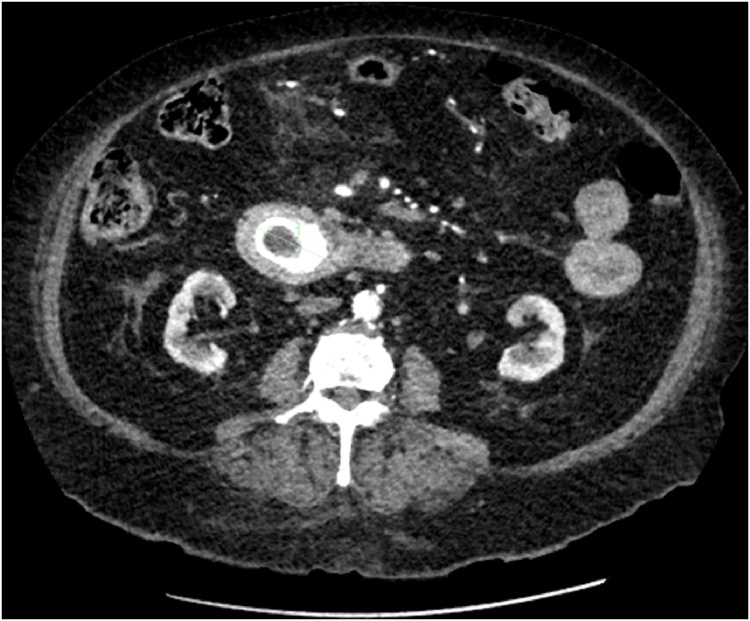
Showing 4.2 cm calcified gallstone within the duodenum on CT abdomen.

**Fig. 2:**
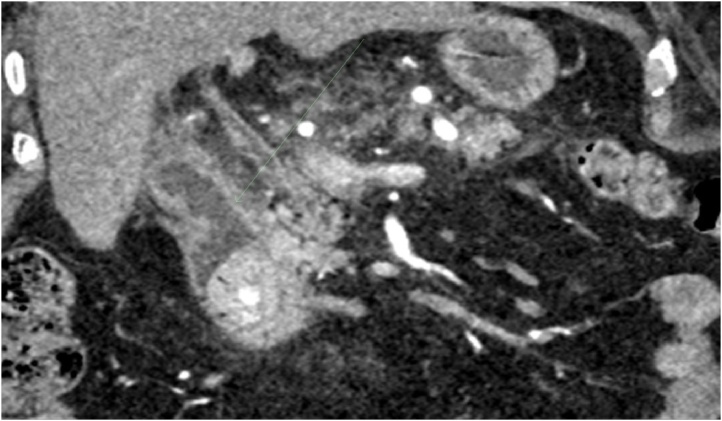
Showing the formed fistula tract between gallbladder and duodenum by the large gallstone on CT abdomen.

**Fig. 3:**
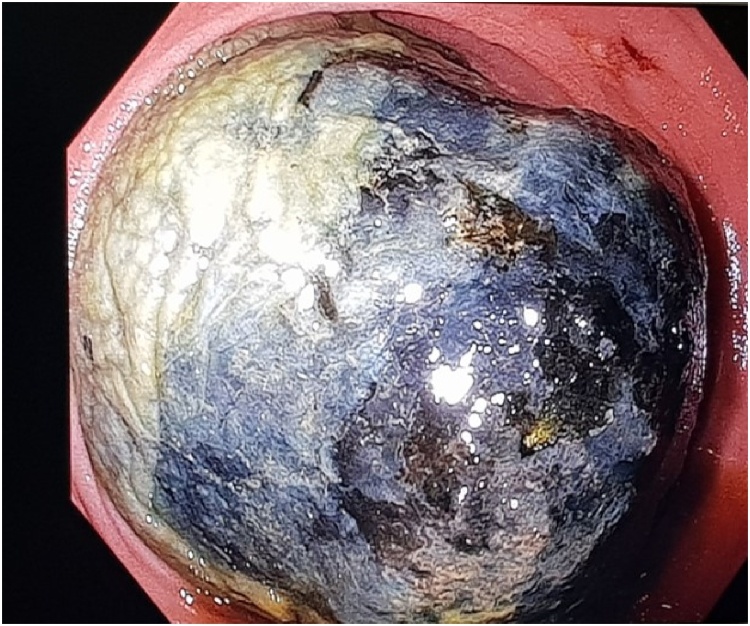
Showing the gallstone in duodenum visualized using endoscopy.

**Fig. 4:**
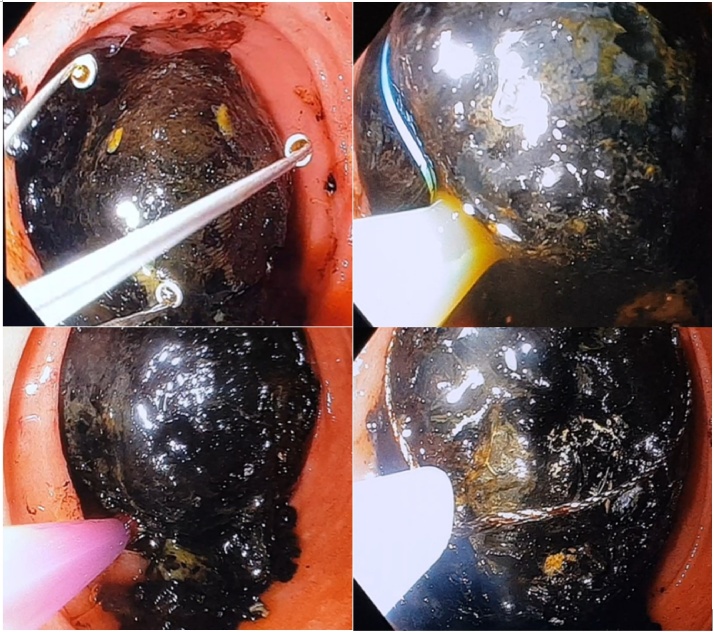
Showing series of endoscopic tools used to retrieve the gallstone.

The patient was admitted under the surgical team for treatment of gallstone pancreatitis. She then had episodes of coffee ground vomitus. Due to persistent nausea and vomiting with high nasogastric tube output, a CT abdomen was performed. The CT revealed that the calculi had fistulised into the duodenum causing the duodenal obstruction as well as associated pneumobilia. There was also presence of peripancreatic inflammation confirming acute pancreatitis (Fig. 1, Fig. 2, Fig. 3, Fig. 4).

An on-table gastroscopy was performed in an attempt to retrieve the impacted stone endoscopically prior to committing to duodenotomy. The gallstone was seen endoscopically to be lodged in third part of duodenum. Unable to be retrieved after using various snares, endoloops, and balloon catheters, a longitudinal duodenotomy was performed at D2/D3 to retrieve the impacted gallstone via right subcostal rooftop incision. The surgery was done by the treating hepatobiliary consultant. The enterotomy site was closed longitudinally with multiple interrupted 3.0 prolene sutures with a patch using transverse colon epiploic appendage. Transverse duodenoplasty was not possible as a result of extensive inflammation due to acute severe pancreatitis. Cholecystectomy and closure of cholecystoduodenal fistula were not performed for this case. A naso-jejunal tube was inserted intraoperatively for enteral feeding.

The patient was admitted into ICU post-operatively for vasopressor support. Assessment of enterotomy repair site with oral contrast was done Day7 post operatively and patient’s enteral diet was subsequently restarted. Patient required rehabilitation and was discharge after 4 weeks of hospitalisation.

Patient has returned to her state of health upon follow up in clinic, 3 months post admission.

## 3 Clinical discussion

First-line treatment of Bouveret’s syndrome tends to be trial of endoscopic stone retrieval [4]. Patients with Bouveret’s syndrome are usually elderly with significant comorbidities, surgical stone retrieval carries the risk of mortality of up to 12% while retrieval with definitive cholecystectomy and fistula closure can be up to 30% [3]. This overall risk is further increased when it is associated with pancreatitis as closure of duodenotomy is even more challenging intraoperatively with its associated increased risk of major complications.

With larger stones not able to be retrieved with simple nets/baskets, lithotripsy can be considered as an adjunct prior to extraction but requires specialized gastroenterology services which may not be readily available. Use of lithotripsy also runs the risk of subsequent gallstone ileus downstream if fragments are not completely removed [13]. Although endoscopic stone retrieval is first-line therapy, its success rate is rather poor at 9%, majority of cases would eventually require surgical stone retrieval [14]. Similar to other known published cases of Bouveret’s syndrome with pancreatitis, surgical extraction of the impacted gallstone was required in this case [8,10,11].

Performing concurrent cholecystectomy with fistula repair remains debatable, requiring consideration of patient’s surgical risk versus risk of gallstone complications versus risk of malignancy [4]. Post-operative management of this case had added challenges due to concurrent systemic inflammatory response from pancreatitis and its burden on the patient’s cardiorespiratory functions, hence a prolonged admission was required.

## 4 Conclusion

Bouveret’s syndrome is a rare form of gallstone ileus which can present concurrently with acute pancreatitis. High suspicion of it is required, especially when patients with gallstone pancreatitis also presents with signs of gastric outlet obstruction. CT imaging or endoscopy should be performed when such symptoms are present to diagnose Bouveret’s syndrome. Endoscopy can be both diagnostic and therapeutic as endoscopic retrieval is usually first-line therapy. However, majority of cases would require surgical extraction as the stones are too large or too impacted to be retrieved endoscopically. Concurrent cholecystectomy with fistula repair is not routinely performed and needs to be assessed individually. The presence of pancreatitis further prolonged the patient’s hospitalization due to management of concurrent systemic inflammatory response from pancreatic injury.

## Declaration of Competing Interest

n/a

## Funding

n/a

## Ethical approval

n/a

## Consent

Written informed consent was obtained from the patient for publication of this case report and accompanying images. A copy of the written consent is available for review by the Editor-in-Chief of this journal on request.

## Author contribution

Wei Shearn Poh: Collated data and imaging, written the paper, and conducted a literature review on Bouveret’s syndrome, including literatures involving Bouveret’s syndrome and pancreatitis.

Ruwan Wijesuriya: Supervisor and reviewed the case report prior to final submission for editorial review and publication.

## Registration of research studies

Not applicable.

## Guarantor

Dr Ruwan Wijesuriya.

## Provenance and peer review

Not commissioned, externally peer-reviewed.
